# Autosomal Recessive Bestrophinopathy: Clinical and Genetic Characteristics of Twenty-Four Cases

**DOI:** 10.1155/2021/6674290

**Published:** 2021-04-30

**Authors:** Hassan Khojasteh, Mohsen Azarmina, Nazanin Ebrahimiadib, Narsis Daftarian, Hamid Riazi-Esfahani, Houra Naraghi, Hamideh Sabbaghi, Alireza Khodabande, Hooshang Faghihi, Afrooz Moghaddasi, Fatemeh Bazvand, Masoud Reza Manaviat, Hamid Ahmadieh, Narges Hassanpoor, Fatemeh Suri

**Affiliations:** ^1^Retina & Vitreous Service, Farabi Eye Hospital, Tehran University of Medical Sciences, Tehran, Iran; ^2^Ophthalmic Research Center, Shahid Beheshti University of Medical Sciences, Tehran, Iran; ^3^Ocular Tissue Engineering Research Center, Shahid Beheshti University of Medical Sciences, Tehran, Iran; ^4^National Institute of Genetic Engineering and Biotechnology, Tehran, Iran; ^5^Department of Ophthalmology, Shahid Sadoughi University of Medical Sciences, Yazd, Iran; ^6^Department of Ophthalmology, Tabriz University of Medical Sciences, Tabriz, Iran

## Abstract

**Background:**

To describe ocular manifestations, imaging characteristics, and genetic test results of autosomal recessive bestrophinopathy (ARB). The study design is an observational case series.

**Methods:**

Forty-eight eyes of 24 patients diagnosed with ARB underwent complete ophthalmic examinations including refraction, anterior and posterior segment examination, enhanced depth imaging optical coherence tomography (EDI-OCT), fluorescein angiography (FA), electroretinography (ERG), and electrooculography (EOG). Optical coherence tomography angiography (OCTA) and BEST1 gene sequencing were performed in selected patients.

**Results:**

The age at onset was 4–35 years (mean: 18.6 years). The male-to-female ratio was 0.45. All patients were hyperopic, except one with less than one diopter myopia. EOG was abnormal in 18 cases with near-normal ERGs. Six patients did not undergo EOG due to their young age. Eighteen patients (75%) had a thick choroid on EDI-OCT, of which three had advanced angle-closure glaucoma, 15 patients were hyperopic, and eight of them had more than four diopters hyperopia in both eyes. Macular retinoschisis was observed in 46 eyes of 23 patients (95%) with cysts mostly located in the inner nuclear layer (INL) to the outer nuclear layer (ONL). Of the 18 patients who underwent FA, mild peripheral leakage was seen in eight eyes of four patients (22%). Subfoveal choroidal neovascularization (CNV) was seen in three eyes of two patients (6%) that responded well to intravitreal bevacizumab (IVB). Seven mutations of the bestrophin-1 (BEST1) gene were found in this study; however, only two of them (p.Gly34 = and p.Leu319Pro) had been previously reported as the cause of ARB based on ClinVar and other literature studies.

**Conclusions:**

ARB can be presented with a wide spectrum of ocular abnormalities that may not be easily diagnosed. Pachychoroid can occur alongside retinal schisis and may be the underlying cause of angle-closure glaucoma in ARB. Our study also expands the pathogenic mutation spectrum of the BEST1 gene associated with ARB.

## 1. Introduction

Bestrophin-1 (BEST1) gene mutations may cause a variety of ocular phenotypes called “bestrophinopathies.” Although a variety of clinical presentations have been described in the literature [1], five known bestrophinopathies include autosomal dominant best or best vitelliform macular dystrophy (BVMD), adult-onset vitelliform macular dystrophy (AVMD), autosomal dominant vitreoretinochoroidopathy (ADVIRC), autosomal recessive bestrophinopathy (ARB), and retinitis pigmentosa (RP). BVMD is the most common phenotype; however, other ocular syndromes such as microcornea, rod-cone dystrophy, early-onset cataract, and posterior staphyloma (MRCS) syndrome are associated with this mutation. Bestrophin-1, the protein product of the BEST1 gene, appears to be multifunctional and plays a role in different intra- and extracellular ionic mechanisms. Ionic metabolism of RPE cells and normal ocular development are adjusted by this protein [2–9].

ARB is caused by mutations in both alleles of the BEST1 gene with either homozygous or compound heterozygous mutations. The clinical features of ARB are more extensive than those of BVMD. These patients have more profound central vision loss with more widespread funduscopic changes and vitelliform material deposition. High hyperopia, shallow anterior segment, angle-closure glaucoma, abnormal or subnormal full-field electroretinography (ERG) (in contrast to normal ERG in BVMD), intraretinal cystic changes, and concurrent subretinal fluid can be seen in ARB [1–6]. In this study, we describe clinical and imaging characteristics of 18 Iranian and six Afghan immigrants diagnosed with ARB. Moreover, choroidal thickness and its association with glaucoma and angle status will be assessed in these patients. Genetic evaluation of 11 patients (from eight families) and their available family members was performed.

To the best of our knowledge, this study is the largest case series of ARB patients worldwide and the first case series from the Middle East.

## 2. Patients and Methods

Patients registered in the Iranian National Registry for Inherited Retinal Diseases who were diagnosed with ARB and ARB patients from Farabi Eye Hospital and Labbafinejad Medical Center from August 2017 to December 2019 were included in this observational case series. The diagnosis was based on clinical characteristics, optical coherence tomography (OCT) abnormalities, electroretinogram (ERG), and electrooculogram (EOG) findings. Written informed consent was obtained from all participants or their guardians before collecting clinical materials and blood samples. This study adhered to the tenets of the Declaration of Helsinki and obtained institutional review board approval. Comprehensive ocular history and ophthalmic examinations, such as refraction, Snellen best-corrected visual acuity (BCVA), slit-lamp anterior and posterior segment examination, gonioscopy, Goldmann applanation tonometry, fundus photography, enhanced depth imaging optical coherence tomography (EDI-OCT), fluorescein angiography (FA), ERG, and EOG, were performed. ERG and EOG were performed according to the International Society for Clinical Electrophysiology of Vision standards and guidelines. Angle closure (AC) was defined as the presence of iridotrabecular contact (ITC) with or without elevated intraocular pressure (IOP) (≥21 mmHg) in the absence of any optic neuropathy. Angle-closure glaucoma was defined as ITC with an elevated IOP and glaucomatous optic neuropathy.

All OCT images were acquired using Heidelberg Spectralis OCT (Heidelberg Engineering, Heidelberg, Germany). In EDI-OCT, subfoveal choroidal thickness >300 *µ*m was considered a thick choroid. Pachychoroid was the term used when a thick choroid was associated with concomitant pachyvessels in EDI-OCT. OCTA was performed in selected patients to rule out subfoveal CNV using Optovue OCTA (Fremont, CA, USA). EOG and ERG were performed by Metrovision (Pérenchies, France) and Retiscan (Roland, Germany, ISCEV standard protocol).

### 2.1. Genetic Analysis Method

Blood samples of 11 patients from eight families and their available family members were collected for genetic evaluation. Genomic DNA was extracted from peripheral blood leukocytes using the standard salting-out protocol. Primers for all coding exons and their flanking regions of the BEST1 gene were designed using GeneRunner version 3.05 software. The primers were checked for specificity to the target sequences of the human genome using NCBI Primer-Blast (http://www.ncbi.nlm.nih.gov/tools/primer-blast/). Primer sequences were available upon request. Polymerase chain reaction (PCR) was performed to amplify the target sequences. Subsequently, all PCR products were sequenced with the Sanger protocol using ABI BigDye Terminator chemistry with an ABI 3730XL genetic analyzer instrument (Applied Biosystems, Foster City, CA, USA). Sequences were analyzed using Sequencher 5.0 software (Gene Codes Corporation, Ann Arbor, MI, USA) and were aligned to the reference sequence of BEST1 (NG_009033.1, NM_004183.4, and NP_004174.1) to determine sequence variations.

### 2.2. Bioinformatics Analysis Method

The pathogenicity of the genetic variants was evaluated using ClinVar (http://www.ncbi.nlm.nih.gov/clinvar/) and the human gene mutation database (http://www.hgmd.cf.ac.uk/ac/index.php) (Table 1). The allele frequency of variants with no clinical significance and the Iranome project (800 genomes of the healthy Iranian people project; http://www.iranome.ir/) were checked in the dbSNP database (https://www.ncbi.nlm.nih.gov/snp/). Subsequently, the combined annotation-dependent depletion (CADD) score (http://cadd.gs.washington.edu/info) was used to predict the pathogenicity of the identified variants. Finally, the candidate causing variation in each patient was screened for segregation within the family by Sanger sequencing. Consequently, variants with minor allele frequency (MAF) < 0.01, according to the reported MAF in the dbSNP database in addition to Iranome and CADD score more than 15, which was segregated with the disease within the families, were considered as likely pathogenic variants for the investigated patients (Table 2). All identified causative variants were checked for the associated phenotypes reported in the publications as well as in the ClinVar database (Table 1).

## 3. Results

**Figure 1:**
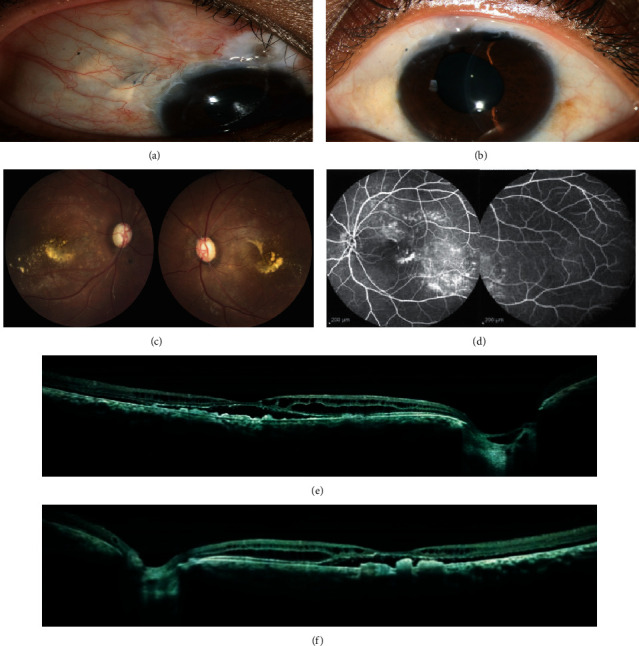
Slit photograph of right (a) and left (b) eyes of patient number 1. She underwent surgical peripheral iridotomy (PI) and trabeculectomy in her both eyes for angle-closure glaucoma. Fundus photo of right and left eyes (c) shows total cup-to-disc ratio and yellow subfoveal materials. Fluorescein angiography of left eye with peripheral views (d) shows staining of yellow vitelliform materials without peripheral leakage. Optical coherence tomography (OCT) of right (e) and left (f) eyes show subfoveal hyporeflective space in both eyes with elongated photoreceptors and intraretinal schisis cavities in inner nuclear layer (INL) and outer nuclear layer (ONL). There are also deposits of debris in the subretinal space mimicking RPE hypertrophy in both eyes.

Twenty-four patients from 20 families were included (18 Iranian and six Afghan immigrants) in this study. The demographic and clinical data of all subjects are summarized in Table 3. At the time of presentation, patients were 4–35 years old (mean: 18.6 years). The male-to-female ratio was 0.45. All patients, except one with less than one diopter myopia, were hyperopic. The most hyperopic refraction was OD: +7.5–1.00 × 30 and OS:  +7.25–1.00 × 150 in patient 7 who was 6.5 years old. BCVA ranged from 40/200 (2/10) to 20/20 (10/10) in the better eye, and only one patient (4.1%) had less than 20/60 visual acuity (low vision cutoff by the World Health Organization) in her better eye. This patient underwent multiple surgeries and ended up with advanced angle-closure glaucoma (Figure 1). The history of strabismus was not reported in any of our patients.

**Figure 2:**
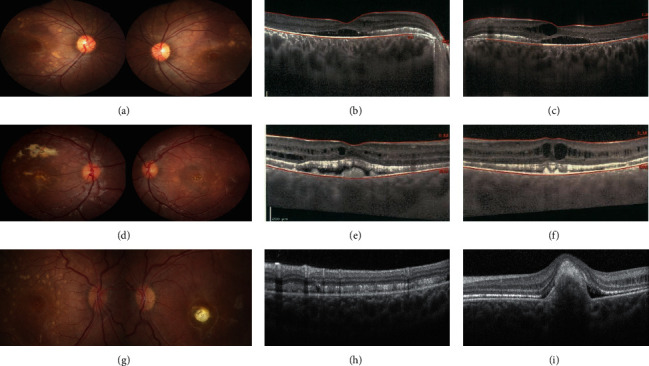
Fundus photo (a, d, g) and pachychoroid in enhanced depth imaging optical coherence tomography (EDI-OCT) (b, c, e, f, h, i) of three patients. Patient no. 8 (first line) was a 16-year-old female with significant pachychoroid in EDI-OCT (b, c) that was diagnosed with angle closure in both eyes in gonioscopy without glaucoma. Patient no. 6 (second line) is an 11-year-old female with high hyperopia that shows significant pachychoroid in EDI-OCT (e, f). She also was diagnosed with angle closure in both eyes in gonioscopy without glaucoma. Patient no. 4 (last line) also shows pachychoroid changes in EDI-OCT (g, i). She also shows a fibrotic pillar in left eye OCT (i).

Retinal lesions varied from bilateral (both symmetric and asymmetric) subfoveal yellow vitelliform materials with and without choroidal neovascularization (CNV) and subretinal fibrosis to multiple small (less than 400 microns in diameter) round yellowish subretinal materials around the arcades and in the macular region (e.g., patient 4, Figure 2(g)). Four patients (patients 4, 5, 14, 15, and 21) showed subretinal fibrosis in the foveal or parafoveal region without any evidence of previous CNV; based on OCT, three of them were compatible with fibrotic pillars (patients 4, 15, and 21; Figures 2(g) and 2(i)). Patient 15 showed amblyopia and sensory esotropia in the eye with subfoveal fibrosis. During a mean follow-up period of 3 years, patients did not show any significant difference in their clinical picture. After recalling all patients for genetic testing, only 11 patients (from 8 families) underwent genetic testing (Table 1). The diagnosis of ARB in other patients was based on clinical findings, multimodal imaging features, and electrophysiological tests.

Twenty-eight eyes (14 patients, 58%) had shallow anterior chambers, three patients did not have respected data documented in their records, and five patients did not undergo angle examination. Three patients had advanced angle-closure glaucoma (patients 1 and 2 who were siblings and patient 13). One of the siblings (the younger one, patient 1) underwent trabeculectomy twice in each eye and shunting procedure in her right eye (Figure 1). The other sibling had controlled IOP in both eyes by laser peripheral iridotomy (PI) and full topical medications (combined timolol/dorzolamide, brimonidine, and latanoprost). Six patients (12 eyes, 25%) underwent treatment for glaucoma.

Thirty-six eyes of 18 patients (75%) were revealed to have thick choroids on EDI-OCT (Table 3, Figure 2), three of which (6 eyes) had advanced angle-closure glaucoma, and the other 15 patients were hyperopic (eight were more than four diopter hyperopic in both eyes).

Forty-six of 48 eyes (95%) showed subretinal hyporeflective space in the fovea and elongated photoreceptors on OCT.

Retinoschisis was observed in 46 of 48 eyes (95%), and cysts were mostly in the inner nuclear layer (INL) to the outer nuclear layer (ONL). All eyes with pachychoroid showed retinoschisis and cystic changes in retinal layers; however, there were four patients (eight eyes) who showed retinoschisis without pachychoroid in their EDI-OCT.

FA showed staining of vitelliform materials and subretinal scars in all patients who underwent angiography without any leakage in the macula secondary to the retinoschisis cavities. Subfoveal CNV was seen in three eyes (two patients) that responded well to intravitreal bevacizumab (IVB) injection. One eye of patient 3 had subfoveal CNV with fluorescein leakage that was difficult to differentiate from adjacent staining of the vitelliform materials; OCTA was helpful in confirming CNV in this patient. In patient 2 who had CNV, OCTA demonstrated a fine vascular tuft in the outer retina. After receiving IVB, subretinal and intraretinal spaces remained stable in both patients; however, regression of small branches was observed on OCTA.

Mild peripheral angiographic leakage was seen in both eyes of 4 of 18 patients (eight eyes, 22%) who underwent FA, all in the temporal quadrants.

The EOG was abnormal in all patients. Five patients who were under 7 years old and one 14-year-old immigrant patient did not undergo EOG. Twenty-nine eyes of 16 patients (80.5% of eyes with calculated EOG) had an Arden ratio less than 120 in EOG with near-normal ERG. One patient had moderately reduced cone and rod responses (patient 9), and four patients had mildly reduced responses in both eyes (patients 8, 16, 18, and 24).

Most patients were misdiagnosed before being referred to tertiary referral centers (18 patients, 36 eyes, 75%). The most common misdiagnose was central serous chorioretinopathy (CSCR), followed by fundus flavimaculatus for multifocal cases (patients 4 and 12). One patient with persistent subretinal fluid, pachychoroid in EDI-OCT, and abnormally dilated vessels in ICG underwent photodynamic therapy with the diagnosis of chronic CSCR. Patient 5 was followed up for several months with an unspecified diagnosis of macular dystrophy before being referred to us. Patient 6 was misdiagnosed with X-linked retinoschisis (XLR) due to OCT abnormalities.

### 3.1. Genetic Data

In total, seven distinct disease-causing variants of the BEST1 gene in eight investigated ARB families were detected, which included five missense (p.Arg13Cys, p.Pro101Leu, p.Gly135Asp, p.Pro297Ser, and p.Leu319Pro), one synonymous (p.Gly34 = ), and one in-frame deletion (p.Gln96_Asn99del). Of these variants, four families carried mutations in exon 4 (4/8,50%), two families in exon 2 (2/8, 25%), one in exon 8 (1/8, 12.5%), and one in exon 9 (1/8, 12.5%) of the BEST1 gene (Table 1). Two of the seven identified mutations in our study (p.Gly34 = and p.Leu319Pro) have been reported as causes of ARB. Mutations p.Gln96_Asn99del and p.Pro101Leu have been previously found to cause RP and autosomal recessive retinal dystrophy, respectively. Heterozygous p.Arg13Cys and p.Pro297Ser mutations have been reported to cause BVMD. There was no phenotype-related report for p.Gly135Asp. All the bioinformatics criteria and segregation analysis of the p.Gly135Asp mutation in patients' family members with this variant confirmed the pathogenicity of this variation.

## 4. Discussion

Diagnosed ARB patients in this study were characterized by early-onset macular edema, serous retinal detachment, diffuse intraretinal schisis cavities, multifocal subretinal vitelliform deposits, abnormal visual electrophysiology, and young age at onset. Most patients had hyperopia and shallow anterior chambers, all of which were consistent with previous reports [1–4]. To the best of our knowledge, this study is the largest case series of ARB patients worldwide and the first case series from the Middle East.

Pathogenic variants of BEST1 result in functional disorder of the RPE, with resultant accumulation of fluid, bis-retinoid N-retinyl-N-retinylidene ethanolamine (A2E), and lipofuscin, which subsequently lead to RPE atrophy and photoreceptor cell loss. As a result, RPE irregularity, macular edema, serous retinal detachment, and vitelliform deposits can be observed in the fundus. Dysfunction of bestrophin can cause maldevelopment of both anterior and posterior segments of the eye characterized by short axial length (AL), hyperopia, and shallow anterior chamber depth (ACD) [3]. Recently, Luo et al. described clinical manifestations and novel genetic mutations in 21 ARB-diagnosed patients in the Chinese population [4] and reported 20 patients with serous retinal detachments, which correlates with our data (48 eyes, 95%). They reported fine peripheral leakage on FA in 47% of patients, while this was observed in only four patients (eight eyes, 22%) in our study. Boon et al. reported peripheral vascular leakage with simultaneous cystoid macular edema in a 28-year-old female with genetically proven ARB that was misdiagnosed as chorioretinitis and was treated with topical and systemic steroids [2], who also had reported patchy deep hyperfluorescent areas on posterior pole angiography. Vasculitis and ocular inflammation can also occur in other congenital retinal dystrophies such as retinitis pigmentosa (RP). Luo et al. [4] reported angle closure/angle-closure glaucoma in 14 patients (66%), which is higher than our results of 58% for angle closure and 25% for angle-closure glaucoma. This might be due to ethnic differences and a higher prevalence of angle-closure glaucoma in East Asia [10]. Generally, a prevalence of 42.8–50% for glaucoma has been reported among ARB patients [4,6,11].

The underlying pathogenesis for angle closure may be due to shallow ACDs and short ALs resulting from the maldevelopment of the eyes secondary to the BEST1 mutation. Alternatively, it may be related to increased choroidal thickness that was present in all eyes with ACG in this study. As ARB patients are prone to develop a flat anterior chamber after trabeculectomy [11], abnormal ciliary body morphology secondary to pachychoroid might be the underlying mechanism.

### 4.1. Pachychoroid in ARB Patients

Luo et al. [4] reported dilated choroidal vessels and choroidal vascular hyperpermeability on ICGA in 25 eyes of 13 patients with ARB. In our case series, 36 eyes of 18 patients (75% of eyes) showed thick choroid and pachyvessels on EDI-OCT (Table 3 and Figure 2), three of which were the same patients with advanced angle-closure glaucoma, and the other 15 patients were hyperopic. Pachychoroid changes in ARB patients can occur as a result of short AL and probably thick sclera in these patients, similar to patients with high hyperopia and nanophthalmos spectrum. Luo et al. hypothesized that the underlying cause of other manifestations of ARB may be choroidal vascular abnormalities that can cause secondary RPE dysfunction and subretinal fluid accumulation [4]. In our series, all cases with pachychoroid showed retinoschisis and cystic changes in retinal layers. In their series of 63 eyes of 51 autosomal dominant bestrophinopathy patients, Grenga et al. concluded there might be a possible pathogenic role for choroid in different stages of the disease [5]. They showed even in adult-onset foveomacular vitelliform dystrophy higher choroidal thickness was associated with the presence of RPE bumps and subretinal fluid [5]. Additional research in this regard is needed.

### 4.2. Retinoschisis in ARB Patients

We found retinoschisis unrelated to CNV in 46 eyes of 23 patients (95%), and cysts were mostly in the INL to the ONL.

Xu et al. reported obvious retinoschisis-like changes in 4 of 10 eyes (40%) with the diagnosis of multifocal vitelliform macular dystrophy [12]. There is a report of a novel mutation in the BEST1 gene in two patients with retinoschisis-like changes, while no mutation was detected in the XLR gene [13].

These cystic changes could be due to structural abnormalities secondary to Muller cell ionic transportation dysfunction and/or disrupted blood ocular barrier secondary to RPE cell ionic transportation dysfunction [2]. One of our cases was misdiagnosed with XLR before being referred to our center. As the appearance of cysts is very similar to XLR with parallel vertical borders, Muller cell dysfunction might have a significant role in combination with other possible causes such as pachychoroid abnormality and RPE dysfunction. Boon et al. reported a significant early improvement of cystoid macular changes in an ARB patient with acetazolamide (250 mg three times a day) [2]. The patient also showed a marked improvement in visual acuity. A similar response to oral or topical carbonic anhydrase inhibitors can be observed in cystoid macular changes associated with other retinal dystrophies, such as XLR and RP [14–17]. None of the patients in this series received oral or topical carbonic anhydrase inhibitors. None of the patients in this series received oral or topical acetazolamide. Although there is not any report regarding intravitreal steroid injection in ARB patients with cystoid foveal changes, there are similar case series in retinitis pigmentosa patients with cystoid macular edema that did not show statistically significant change in BCVA [18]. None of the patients in this series received intravitreal steroid injection.

### 4.3. ARB Misdiagnosis

In this case series, most patients (75%) were misdiagnosed before referring to our tertiary centers. This might be due to the rarity of this disease and previously unreported clinical features of this entity. The most common incorrect impressions were CSCR, followed by fundus flavimaculatus, unspecified macular dystrophy, and XLR. An erroneous diagnosis of chorioretinitis has been reported by Boon. Definite diagnoses are based on genetic analysis. Clinical and paraclinical features that may guide clinicians to the correct diagnosis and avoid unnecessary treatments include early onset bilateral vitelliform depositions, cystic changes in retinal layers, hyperopia, shallow ACD, ACG, and abnormal EOG with mild to moderate abnormality in the ERG.

### 4.4. Genetic Evaluation of ARB Patients

Our work expands the pathogenic mutation spectrum of BEST1 associated with ARB and the clinical features of the disease, leading to improved diagnosis and genetic counseling criteria for affected patients and their families. In our study, missense mutations were the leading cause of ARB (5/7, 71%), which is in concordance with other reported cases [19–23].

In total, seven mutations of BEST1 were found in this study; however, only two of them (p.Gly34 = and p.Leu319Pro) had been previously reported as the cause of ARB based on ClinVar and other literature studies. Two other mutations have been introduced as the cause of RP (p.Gln96_Asn99del) and autosomal recessive retinal dystrophy (p.Pro101Leu), showing variable clinical manifestations of the same mutations in different individuals. Heterozygous p.R13C and p.Pro297Ser have been reported for BVMD, but we discovered for the first time both of the mutations in homozygous states in our ARB patients, suggesting that the mutational spectrum of autosomal recessive and autosomal dominant forms of bestrophinopathies could overlap, and other heterozygous carriers of the ARB-affected families may require regular ophthalmic examinations and genetic counseling. There were no reported phenotypes associated with p.Gly135Asp variation and this was identified for first time as the cause of ARB (Table 1).

Limitations of this study include the lack of genetically proven diagnosis of ARB and data regarding AL in all patients.

## 5. Conclusions

In this case series of 24 patients with ARB, the majority had pachychoroid associated with angle closure/angle-closure glaucoma. All patients with pachychoroid showed retinoschisis and intraretinal cystic changes. A high index of suspicion is needed for the correct diagnosis of ARB.

## Figures and Tables

**Table 1:** BEST1 mutations of genetic testing results.

Family	Patient no.	Position	Nucleotide change	Amino acid change	Zygosity	Mutation type	SNP ID	Reported clinical significance/reported phenotype (based on ClinVar)	Associated disease in publications
1	11	Exon 2	c.37 C > T	p.R13 C	Homo	Missense	rs886041141	Not provided/—	BVMD (Ref. [21])
2	7	Exon 2	c.102 C > T	p.Gly34 =	Homo	Synonymous	rs771898125	Likely pathogenic/—	ARB (Ref. [22])*∗*
2	22	Exon 2	c.102 C > T	p.Gly34 =	Homo	Synonymous	rs771898125	Likely pathogenic/—	ARB (Ref. [22])*∗*
3	17	Exon 4	c.287_298del	p.Gln96_Asn99del	Homo	In-frame deletion	rs1555099048	Likely pathogenic/RP	—
4	23	Exon 4	c.302 C > T	p.Pro101Leu	Homo	Missense	rs374517178	Not provided/—	Autosomal recessive retinal dystrophy (Ref. [23])*∗*
5	24	Exon 4	c.404 G > A	p.Gly135Asp	Homo	Missense	rs1159966472	Not provided/—	—
6	4	Exon 4	c.404 G > A	p.Gly135Asp	Homo	Missense	rs1159966472	Not provided/—	—
7	21	Exon 8	c.889 C > T	p.Pro297Ser	Homo	Missense	rs1805143	Likely pathogenic/BVMD	—
7	20	Exon 8	c.889 C > T	p.Pro297Ser	Homo	Missense	rs1805143	Likely pathogenic/BVMD	—
8	19	Exon 9	c.956 T > C	p.Leu319Pro	Homo	Missense	rs1554963305	Pathogenic/ARB	—
8	18	Exon 9	c.956 T > C	p.Leu319Pro	Homo	Missense	rs1554963305	Pathogenic/ARB	—

ARB: autosomal recessive bestrophinopathy; BVMD: best vitelliform macular dystrophy. *∗*Reference number for the publication.

**Table 2:** Pathogenicity criteria for identified variants with no provided clinical significance.

SNP ID	Highest population MAF	Frequency in Iranome	CADD score	Segregation in the family
rs886041141	<0.01	None	22.2	Yes
rs374517178	<0.01	None	32	Yes
rs1159966472	<0.01	None	24.4	Yes

SNP ID: single-nucleotide polymorphism database identification; MAF: minor allele frequency; CADD score: combined annotation-dependent depletion score.

**Table 3:** Demographic and clinical data of all subjects.

Case no.	Age (y)/sex	BCVA	Cyclorefraction	Arden ratio	ERG	Thick choroid	Retinal schisis unrelated to CNV/involved layers	Subretinal hyporeflective space in fovea in OCT	ACG treatment	CNV	Fine peripheral leakage in FA	Genetically proven
1	20/F	2/10 1/10	+2.00–2.5 × 100 +3.00–2.5 × 75	101 107	NL	OU	OU/INL > ONL	OU	+(Surgical PI OU/TBX OU × 2/AGV OD)	—	—	—
2	27/M	6/10 8/10	+2.00–1.00 × 90 +2.50–1.50 × 85	112 113	NL	OU	OU/INL	OU	+Zilomole-brimonidine-latanoprost OU/laser PI OU	OD, IVB × 1	—	—
3	25/M	5/10 2/10	−0.5−0.5 × 180 P−0.75 × 30	118 115	NL	—	OU/INL	OU	—	OU, IVB × 3	—	—
4	12/F	7/10 3/10	+4.5–0.5 × 50 +5.00–0.5 × 110	102 102	NL	OU	OU/INL	OU	—	- (Subfoveal scar in OS compatible with fibrotic pillar)	—	+
5	14/M	5/10 5/10	P-0.75 × 10 +0.50–1.5 × 90	111 107	NL	_	OU/INL	OU	—	-(Old subfoveal scar in OD)	—	—
6	11/F	6/10 4/10	+4.75–0.25 × 90 +5.75–0.5 × 90	107 102	NL	OU/severe	OU/INL > ONL	OU	—	—	—	
7	6.5/F	8/1 4/10	+7.5–1.00 × 30 +7.251.00 × 150	NA due to poor cooperation	NL	OU	OU/ INL > ONL	OU	—	—	NA	
8	16/F	4/10 7/10	+1.00–1.00 × 5 +1.25–0.75 × 175	103 103	Mildly reduced photopic and scotopic responses	OU, severe	OU/INL	OU	—	—	—	—
9	30/M	1/10 3/10	+1.00 +1.00–0.5 × 180	168 170	Moderately reduced photopic responses	—	OU/INL, ONL CME like pattern	—	—	—	+	—
10	34/M	5/10 6/10	+2.75–0.5 × 90 +2.25–0.75× 90	113 108	NL	—	—	OU	—	—	—	—
11	10/F	7/10 6/10	+2.75–0.5 × 180 +2.5–1.00 × 180	101 103	NL	—	OU, INL	OU	—	—	—	+
12	33/M	6/10 5/10	+1.75–1.25 × 10 +2.00–0.75 × 170	103 107	NL	OU	OU, INL	OU	—	—	—	—
13	35/F	6/10 6/100	+2.75 +1.00	111 106	NL	OU, severe	OU, INL > ONL	OU	+ (Laser PI, OU/cataract surgery OU/ zilomole-brimonidine-latanoprost OU)	—	—	—
14	4/M	Poor cooperation	+5.00–1.5 × 20 +5.5–0.75 × 180	NA due to poor cooperation	NA	NA	OU, INL	OU	—	-(OS: subfoveal fibrosis)	NA	—
15	7/M	1/10 7/10	+4.75 +4.5–0.5 × 180	NA due to poor cooperation	NA	OU	OU/INL, ONL	OU	—	_ ((Subfoveal scar in OD compatible with fibrotic pillar)	NA	—
16	14/F	8/10 8/10	+4.75–0.75 × 180 +4.00	NA due to poor cooperation	Mildly reduced responses	OU	OU/INL, ONL	OU	—	—	NA	—
17	32/M	2/10 2/10	+2.75–0.75 × 180 +2.50	108 112	NL	OU, severe	OU/INL, ONL	OU	+ (OU/latanoprost)	—	+	+
18	28/F	10/10 6/10	+1.5–0.25 × 60 +1.50	132 115	Mildly reduced responses	OU, severe	OU/INL, ONL	OU	+ (OU/laser PI)	—	+	+
19	26/F	5/10 10/10	+0.75–1.25×10 +0.5–0.75×180	176 189	Near-normal responses	OU	OU/INL, ONL	OU	—	—	+	+
20	7/F	6/10 6/10	+5.75–1.25 × 10 +5.75–1 × 170	NA due to poor cooperation	NL	OU, severe	OU. INL	OU	—	—	NA	+
21	12/M	2/10 4/10	+3.50–3.00 × 170 +2.75–2.00 × 170	110 124	NL	OU	OU/INL, ONL	OU	—	_ (Subfoveal scar in OD compatible with fibrotic pillar)	—	+
22	5/F	7/10 6/10	+4.5 +4.25–0.75 × 60	NA due to poor cooperation	NL	OU	OU, INL	OU	—	—	NA	+
23	11/F	7/10 5/10	+3.50–3.00 × 180 +2.75–1.25 × 180	121 117	NL	OU	OU/INL, ONL	OU	+ (OU/timolol)	—	—	+
24	27/M	5/10 5/10	+1.50–0.75 × 180 +1.5–0.5 × 180	108 105	Mildly reduced responses	OU	OU/INL, ONL	OU	—	—	—	+

BCVA: best-corrected visual acuity; OU: both eyes; ACG: angle-closure glaucoma; PI: peripheral iridotomy; TBX: trabeculectomy; IOP: intraocular pressure; OCT: optical coherence tomography; INL: inner nuclear layer; ONL: outer nuclear layer; CME: cystoid macular edema; FA: fluorescein angiography; ERG: electroretinogram; NL: normal; NA: not available; F: female; M: male; CNV: choroidal neovascularization.

## Data Availability

The data used to support the findings of this study will be available upon request.

## References

[1] Toto L. L., Boon C. J., Di Antonio L. (2016). BESTROPHINOPATHY: a spectrum of ocular abnormalities caused by the c.614T > C mutation in the BEST1 gene. *Retina*.

[2] Boon C. J., Van Den Born L. I., Visser L. (2013). Autosomal recessive bestrophinopathy: differential diagnosis and treatment options. *Ophthalmology*.

[3] Spaide R. (2008). Autofluorescence from the outer retina and subretinal space: hypothesis and review. *Retina*.

[4] Luo J., Lin M., Guo X. (2018). Novel BEST1 mutations and special clinical characteristics of autosomal recessive bestrophinopathy in Chinese patients. *Acta Ophthalmologica*.

[5] Grenga P. L., Fragiotta S., Cutini A., Meduri A., Vingolo E. M. (2016). Enhanced depth imaging optical coherence tomography in adult-onset foveomacular vitelliform dystrophy. *European Journal of Ophthalmology*.

[6] Boon C. J., Klevering B. J., Leroy B. P. (2009). The spectrum of ocular phenotypes caused by mutations in the BEST1 gene. *Progress in Retinal and Eye Research*.

[7] Davidson A. E., Millar I. D., Urquhart J. E. (2009). Missense mutations in a retinal pigment epithelium protein, bestrophin-1, cause retinitis pigmentosa. *American Journal of Human Genetics*.

[8] Dutta Majumder P., Menia N., Roy R. (2018). Uveitis in patients with retinitis pigmentosa: 30 Years’ consecutive data. *Ocular Immunology and Inflammation*.

[9] Burgess R., Millar I. D., Leroy B. P. (2008). Biallelic mutation of BEST1 causes a distinct retinopathy in humans. *The American Journal of Human Genetics*.

[10] Chan E. W., Li X., Tham Y. C. (2016). Glaucoma in Asia: regional prevalence variations and future projections. *British Journal of Ophthalmology*.

[11] Zhong Y., Guo X., Xiao H. (2017). Flat anterior chamber after trabeculectomy in secondary angle-closure glaucoma with BEST1Gene mutation: case series. *PLoS One*.

[12] Xu H., Ying L., Lin P., Wu J. (2013). Optical coherence tomography for multifocal vitelliform macular dystrophy. *Optometry and Vision Science*.

[13] Silva R. A. L., Berrocal A. M., Lam B. L. (2013). Novel mutation in BEST1 associated with retinoschisis. *JAMA Ophthalmology*.

[14] Grover S., Apushkin M. A., Fishman G. A. (2006). Topical dorzolamide for the treatment of cystoid macular edema in patients with retinitis pigmentosa. *American Journal of Ophthalmology*.

[15] Ghajarnia M., Gorin M. B. (2007). Acetazolamide in the treatment of X-linked retinoschisis maculopathy. *Archives of Ophthalmology*.

[16] Genead M. A., McAnany J. J., Fishman G. A. (2012). Topical dorzolamide for treatment of cystoid macular edema in patients with choroideremia. *Retina*.

[17] Genead M. A., Fishman G. A., McAnany J. J. (2010). Efficacy of topical dorzolamide for treatment of cystic macular lesions in a patient with enhanced S-cone syndrome. *Documenta Ophthalmologica*.

[18] Scorolli L., Morara M., Meduri A. (2007). Treatment of cystoid macular edema in retinitis pigmentosa with intravitreal triamcinolone. *Archives of Ophthalmology*.

[19] Gao T., Tian C., Hu Q. (2018). Clinical and mutation analysis of patients with best vitelliform macular dystrophy or autosomal recessive bestrophinopathy in Chinese population. *BioMedical Research International*.

[20] Tian L., Sun T., Xu K., Zhang X., Peng X., Li Y. (2017). Screening of BEST1 gene in a Chinese cohort with Best vitelliform macular dystrophy or autosomal recessive bestrophinopathy. *Investigative Ophthalmology & Visual Science*.

[21] Dalvin L. A., Pulido J. S., Marmorstein A. D. (2017). Vitelliform dystrophies: prevalence in olmsted county, Minnesota, United States. *Ophthalmic Genetics*.

[22] Davidson A. E., Sergouniotis P. I., Burgess-Mullan R. (2010). A synonymous codon variant in two patients with autosomal recessive bestrophinopathy alters in vitro splicing of BEST1. *Molecular Vision*.

[23] Avela K., Sankila E. M., Seitsonen S. (2018). A founder mutation in CERKL is a major cause of retinal dystrophy in Finland. *Acta Ophthalmologica*.

